# Personalised mapping of tumour development in synchronous colorectal cancer patients

**DOI:** 10.1038/s41525-020-0134-3

**Published:** 2020-07-03

**Authors:** Valentina Thomas, Maura B. Cotter, Miriam Tosetto, Yi Ling Khaw, Robert Geraghty, Desmond C. Winter, Elizabeth J. Ryan, Kieran Sheahan, Simon J. Furney

**Affiliations:** 1 Genomic Oncology Research Group, Department of Physiology and Medical Physics, Royal College of Surgeons in Ireland, Dublin, Ireland; 2 Centre for Systems Medicine, Royal College of Surgeons in Ireland, Dublin, Ireland; 3 Centre for Colorectal Disease, St. Vincent’s University Hospital, Dublin, Ireland; 4 School of Medicine, University College Dublin, Dublin, Ireland; 5 Present Address: Health Research Institute, Department of Biological Sciences, University of Limerick, Limerick, Ireland

**Keywords:** Genome informatics, Colorectal cancer, Cancer genomics

## Abstract

Synchronous colorectal cancers (syCRCs) are two or more primary tumours identified simultaneously in a patient. Previous studies report high inter-tumour heterogeneity between syCRCs, suggesting independent origin and different treatment response, making their management particularly challenging, with no specific guidelines currently in place. Here, we performed in-depth bioinformatic analyses of genomic and transcriptomic data of a total of eleven syCRCs and one metachronous CRC collected from three patients. We found mixed microsatellite status between and within patients. Overlap of mutations between synchronous tumours was consistently low (<0.5%) and heterogeneity of driver events across syCRCs was high in all patients. Microbial analysis revealed the presence of *Fusobacterium nucleatum* species in patients with MSI tumours, while quantification of tumour immune infiltration showed varying immune responses between syCRCs. Our results suggest high heterogeneity of syCRCs within patients but find clinically actionable biomarkers that help predict responses to currently available targeted therapies. Our study highlights the importance of personalised genome and transcriptome sequencing of all synchronous lesions to aid therapy decision and improve management of syCRC patients.

## Introduction

Colorectal cancer (CRC) is the third most frequently diagnosed malignancy and the fourth leading cause of cancer-related deaths worldwide^1^. The main challenge in the treatment of this disease is its high intra- and inter-tumour heterogeneity, which develops through multiple genetic and epigenetic pathways of genome instability, each contributing distinct features to the tumour genome^2–4^. CRCs vary in their cancer-associated driver mutations, which can be found in a number of genes, such as *KRAS* and *BRAF*^5^. About 15% of CRCs acquire abnormalities in DNA mismatch repair (MMR) genes, which lead to microsatellite instability (MSI)^6,7^. MSI is typically mutually exclusive with chromosomal instability (CIN), which accounts for the majority of lesions^8,9^. CRCs also vary in their microbiome composition, with some enriched in *Fusobacterium nucleatum* and decreasing in size after antibiotic treatment^10^. Knowledge on the status of said features in a cancer provides biomarkers that predict its response to targeted therapies, such as *KRAS* wild type status for anti-EGFR therapy, *BRAF* mutant status for combined *BRAF* and *MEK* inhibition therapy, MSI status for immunotherapy, high CIN for VEGF-A combination therapy, and *Fusobacterium*-load for antimicrobial intervention^10–21^. However, as in the case of a subset of *KRAS* wild type tumours that do not respond to anti-EGFR therapy^22^, not all occurrences behave analogously, outlining the need for multiple biomarkers to improve management. Recent research shows that different molecular characteristics, prognosis and treatment outcome of CRC also vary according to tumour sidedness^22^ and tumour immune contexture^23,24^. Further efforts to advance targeted intervention focused on subtyping CRCs based on gene expression profiles and yielded two major classifiers: the consensus molecular subtypes and the CRC intrinsic subtypes, both of which hold significant potential for further diagnostic value^25,26^.

About 4% of CRC patients develop multiple primary colorectal tumours diagnosed simultaneously or within 6 months of each other, known as synchronous CRCs (syCRCs)^27,28^. Predisposing known genetic conditions are causative for about only 10% of syCRCs^27^, suggesting that other genetic and environmental risk factors are involved. Previous studies on syCRCs have reported high heterogeneity of variants between synchronous tumours, with distinct mutations occurring in known CRC genes, and variation between tumour signature content, immune cell scores and MSI status^29–32^. Although prognosis of syCRC patients does not seem to vary significantly from that of solitary CRC patients^30,33^, an understanding of the mechanisms implicated in this phenomenon is still limited and no specific guidelines are currently available for the management and treatment of synchronous cases.

Here, we performed an in-depth characterisation of 12 tumours from 3 syCRC patients (Table 1) by analysing histopathological, whole-genome sequencing (WGS) and RNA-sequencing data. We assessed the extent of genetic overlap between synchronous tumours and examined associations between clinicopathological information and the molecular, microbial and immune features of each tumour genome.

**Table 1:** Clinicopathologic data of patients.

Patient	A	B	C
Gender	Male	Female	Male
Age	36	79	70
Other conditions	Ulcerative colitis	Small bowel carcinoid	Marginal zone lymphoma
Surgery	Subtotal colectomy	Subtotal colectomy	(1) Right hemicolectomy
	End ileostomy formation		(2) Subtotal colectomy
Tumours	A1 (MSS)	B1 (MSI)	C1 (MSI)
	A2 (MSS)	B2 (MSI)	C2 (MSI)
		B3 (MSI)	C3 (MSI)
		B4 (MSI)	C4 (MSI)
		B5 (MSS)	C5 (MSI)
Location	Ascending colon (A1, A2)	Descending colon (B1)	Caecum (C1)
		Hepatic flexure (B2)	Ascending colon (C2, C3, C4)
		Transverse colon (B3)	Sigmoid colon (C5)
		Splenic flexure (B4)	
		Caecum (B5)	
Stage	pT4aN2b (A1)	pT3N1 (B1)	pT4 N0 (C1)
	pT4bN2b (A2)	pT3N1 (B2)	pT2 N0 (C2)
		pT3N1 (B3)	pT3 N0 (C3)
		pT3N1 (B4)	pT2 N0 (C4)
		pT3N1 (B5)	pT3 N0 (C5)
Differentiation	Poor (A1)	Moderate (B1)	Moderate (C1)
	Poor (A2)	Moderate (B2)	Moderate (C2)
		Moderate (B3)	Moderate (C3)
		Moderate (B4)	Moderate (C4)
		Moderate (B5)	Poor (C5)
Mucinous component	<10% (A1)	0% (B1)	0% (C1)
	0% (A2)	40% (B2)	30% (C2)
		60% (B3)	60% (C3)
		70% (B4)	70% (C4)
		10% (B5)	0% (C5)

A total of 12 tumours (11 primary and 1 metachronous) from 3 patients were analysed.

## Results

### Sample description

A total of twelve tumour samples (11 primary and 1 metachronous) were collected from three treatment naive sporadic (i.e., non-hereditary) CRC patients (Patient A, Patient B, and Patient C) and analysed in this study. Clinicopathologic data of patients are provided in Table 1. The patients reported no family history of CRC, and the majority of tumours were located on the right side of the colon but span from the caecum to the sigmoid colon.

### Patient A

**Fig. 1:**
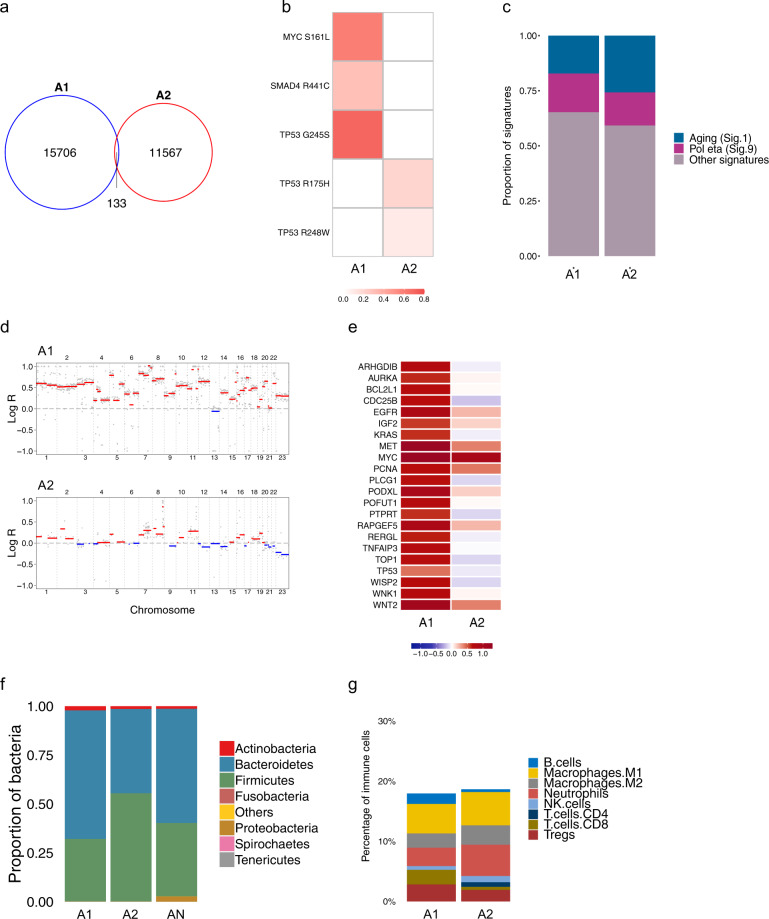
Genomic and transcriptomic analyses for patient A. **a** A Venn diagram of SNVs shows 0.49% overlap between tumours. **b** Variant Allele Frequencies (VAFs) of putative driver mutations show heterogeneous drivers in A1 and A2. **c** Mutational signature analysis. **d** Genomic landscape of CNAs shows CIN in both A1 (ploidy of 3.58) and A2 (ploidy of 2.2). **e** Log-ratio of putative driver CNAs highlights heterogeneity of tumourigenic events between A1 and A2. **f** Microbial analysis of DNA data shows microbial abundance at the phylum level. **g** Quantification of tumour immune infiltration for eight immune cell populations across A1 and A2.

Patient A was a 36-year-old male with a history of ulcerative colitis, including numerous flare-ups over the preceding 2 years and had been treated with Pentasa (mesalazine/5ASA). Background inactive chronic colitis and low-grade dysplasia was identified in almost every section on histological examination, suggesting a field effect across the entire colon. The tumours were therefore arising in a bed of inflammation rather than in normal mucosa. Two primary tumours in the ascending colon were collected (tumours A1 and A2). WGS revealed a higher mutation burden in tumour A1 compared with A2, for all mutation types (Supplementary Table 1). The majority of mutations found in patient A were unique to either A1 or A2 (Fig. 1a and Supplementary Fig. 1a, b). Distinct *TP53* driver mutations were found in both tumours. Additional mutations in *SMAD4* and *MYC* occurred in A1, while none of the overlapping mutations were identified as a known driver (Fig. 1b). This suggests that syCRCs in patient A are genetically distinct and likely to have originated independently. We performed mutational signature analysis (https://cancer.sanger.ac.uk/cosmic/signatures_v2) to investigate the mutational processes that occurred during tumour development. This analysis revealed similar signature profiles in A1 and A2, with a significant proportion of the age-related signature 1. (Fig. 1c and Supplementary Fig. 1c). No MMR-deficiency related signature was found. Copy number alteration (CNA) analysis revealed high CIN in both tumours and tumour A1 appeared to exhibit hyperdiploidy (Fig. 1d and Supplementary Fig. 1d). *EGFR* was amplified in both lesions and the amplification of other known CRC oncogenes, such as *KRAS* and *TOP1*, was exclusive to either A1 or A2. Similarly, *TP53* was found to be deleted in A2 but amplified in A1, further highlighting heterogeneity of these tumours (Fig. 1e).

DNA analysis of gut microbial organisms in patient A revealed high abundance of *Bacteroidetes* and *Firmicutes spp*. (Fig. 1f). Transcriptomic subtyping predicted both tumours as CMS4 and CRIS-B (Supplementary Table 1). A1 displayed lower abundance of neutrophils but greater amounts of B cells and CD8^+^ T cells, whereas CD4^+^ T cells were only detected in A2, which showed higher neutrophil-to-lymphocyte ratio and higher CD4/CD8 ratio, both linked to poor clinical outcomes (Fig. 1g).

### Patient B

**Fig. 2:**
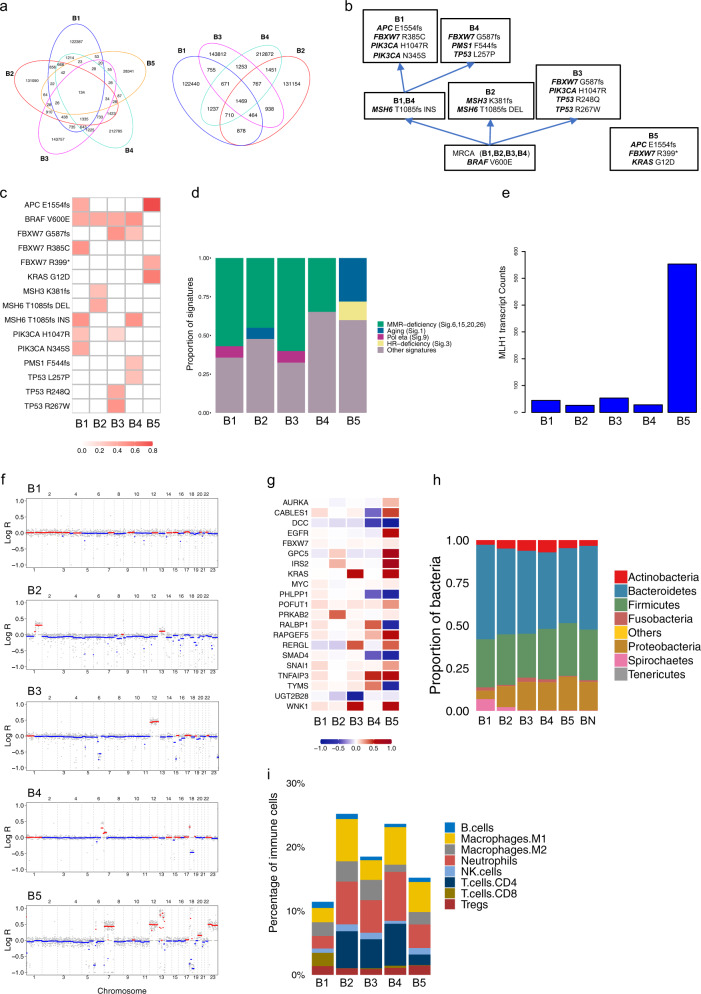
Genomic and transcriptomic analyses for patient B. **a** Venn diagrams of SNVs for all lesions B1–B5 (left) and for MSI lesions B1–B4 (right) show low overlap between tumours. **b** Putative phylogenetic tree based on driver mutations. **c** VAFs of putative driver mutations. **d** Mutational signature analysis. **e** Transcript counts of the *MLH1* detected by RNA-seq analysis. **f** Genomic landscape of CNAs shows low CIN for MSI samples B1 (ploidy: 2), B2 (ploidy: 1.95), B3 (ploidy: 2) and B4 (ploidy: 1.98), and higher CIN for MSS sample B5 (ploidy: 2.13). **g** Median log-ratio of putative driver CNAs highlights heterogeneity of tumourigenic events between all lesions. **h** Microbial analysis of DNA data shows microbial abundance at the phylum level. **i** Quantification of tumour immune infiltration reveals distinct profiles for eight immune cell populations across B1–B5.

Patient B was a 79-year-old female presenting with five primary synchronous tumours ranging from the caecum to the sigmoid colon (tumours B1–B5; Table 1). Higher single-nucleotide variant (SNV) and InDel burdens were found in tumours B1–B4 compared with B5, which, in turn, showed the highest number of structural variants (SVs; Supplementary Table 1). Most mutations found in patient B were unique to each primary tumour (Fig. 2a and Supplementary Fig. 2a, b). However, the same *BRAF* V600E driver mutation was identified in all MSI tumours B1–B4. Tumour B2 further experienced a deletion in the *MSH3* and *MSH6* genes, whereas tumours B1 and B4 showed an *MSH6* insertion, with tumour B1 acquiring further mutations in *PIK3CA* H1047R and *FBXW7* R385C and tumour B4 developing mutations in *FBXW7* G587fs, *PMS1* F544fs and *TP53* L257P. Mutations in *PIK3CA* H1047R and *FBXW7* G587fs were also found in B3. B5 (MSS tumour) shared one *APC* E1554fs mutation with B1, but no mutations with the rest of the primaries, presenting distinct ones: *FBXW7* R399* and *KRAS* G12D (Fig. 2b, c). Driver SNV heterogeneity in the five tumours was corroborated by mutation calling analysis of the RNA-seq data.

Mutational signature analysis showed tumours B1–B4 with a high proportion of MMR-deficiency-related signatures, and absence of these signatures in B5 (Fig. 2d and Supplementary Fig. 2c). This was corroborated by protein immunohistochemistry (IHC), which showed loss of *MLH1* in tumours B1–B4 and reduction in *MLH1* transcript expression (Fig. 2e), likely due to hypermethylation of the *MLH1* promoter. CNA analysis revealed low CIN in MSI tumours (B1–B4) and high CIN in the MSS tumour B5 (Fig. 2f and Supplementary Fig. 2d). B5 was the only sample to show *EGFR* amplification, *KRAS* was amplified in B3 and B5 while tumour suppressor genes, such as *DCC* and *SMAD4*, were deleted in B4 and B5 (Fig. 2g).

DNA analysis of gut microbial organisms associated with each tumour within patient B revealed prevalence of *Bacteroidetes* and *Firmicutes spp*., and evidence of *F. nucleatum* in all lesions (Fig. 2h). Interestingly, tumours B1 and B5 were both categorised as CMS2 and CRIS-E. These are in concordance with the high CIN and *KRAS* mutant state of B5 but not of B1. As expected, B2, B3 and B4 were assigned to the MSI-like and *BRAF*-mutated CRIS-A. B4 was also identified as CMS3 (Supplementary Table 1). The MSS B5 tumour showed an immune infiltration of ~15%, while infiltration in MSI tumours ranged from ~11% in B1 to ~25% in B2. Together with the lowest infiltration, B1 showed a lower abundance of neutrophils, a higher fraction of CD8+ T cells and a lack of CD4+ T cells. The highest neutrophil-to-lymphocyte ratio was seen in B5, followed by B4, B2, B3 and B1 (Fig. 2i).

### Patient C

**Fig. 3:**
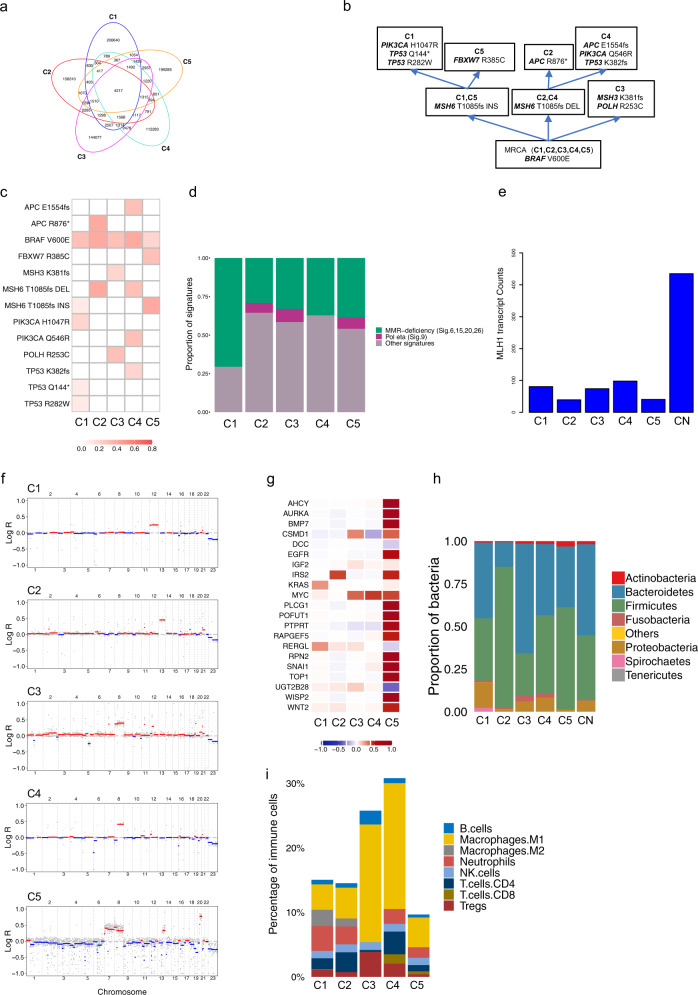
Genomic and transcriptomic analyses for patient C. **a** Venn diagram of SNVs shows low overlap between tumours. **b** Putative phylogenetic tree based on driver mutations. **c** VAFs of putative driver mutations. **d** Mutational signature analysis. **e** Transcript counts of the MLH1 detected by RNA-seq analysis. **f** Genomic landscape of CNAs shows low CIN for all samples C1 (ploidy: 2.03), C2 (ploidy: 2.06), C3 (ploidy: 2.13), C4 (ploidy: 2.02) and C5 (ploidy: 2.13). **g** Median log-ratio of putative driver CNAs highlights heterogeneity of tumourigenic events between all lesions. **h** Microbial analysis of DNA data shows microbial abundance at the phylum level. **i** Quantification of tumour immune infiltration reveals varying fractions for eight immune cell populations across all samples.

Patient C was a 70-year-old male who presented initially with four primary synchronous tumours (C1–C4), and with a metachronous tumour (C5) more than 6 months later. WGS revealed similar burdens of SNVs, InDels and SVs in all tumour samples in patient C (Supplementary Table 1). Most mutations occurred exclusively in each lesion (Fig. 3a and Supplementary Fig. 3a, b). The same *BRAF* V600E mutation occurred in all tumours, identifying it as a likely early event in tumour development (Supplementary Fig. 3c). Tumours C1 and C5 further acquired an insertion in the *MSH6* gene, with tumour C1 acquiring mutations in *PIK3CA* H1047R, *TP53* Q144* and *TP53* R282W, and tumour C5 developing a mutation in *FBXW7* R385C. In contrast, tumours C2 and C4 experienced a deletion in the *MSH6* gene, with tumour C2 acquiring additional mutations in *APC* R876* and tumour C4 gaining mutations in *APC* E1554fs, *PIK3CA* Q546R and *TP53* K382fs. C3 experienced a deletion in *MSH3* and acquired a mutation in *POLH* R253C (Fig. 3b, c and Supplementary Fig. 3d). Driver SNV heterogeneity was corroborated by analysis of the RNA-seq data. Mutational signature analysis unveiled high proportions of MMR-deficiency related signatures in all tumours (Fig. 3d and Supplementary Fig. 3e). This was corroborated by loss of *MLH1* expression detected by IHC and reduction in *MLH1* transcript expression (Fig. 3e) in all tumours. CNA analysis reported low CIN in all tumours in patient C (Fig. 3f and Supplementary Fig. 3f). We identified amplified oncogenes, such as *KRAS* in C1 and *MYC* in C3–C5, and deleted tumour suppressor genes, such as *DCC* in C5 (Fig. 3g).

DNA analysis of gut microbial organisms associated with each tumour revealed prevalence of *Bacteroidetes* and *Firmicutes spp*. across all lesions. DNA evidence of *F. nucleatum* was found in all tumours C1–C5 (Fig. 3h). C1 was classified as CMS4 and CRIS-D. CRIS-D was also assigned to C3, where an amplification of *IGF2* was observed. C2 was classified as CMS2 and CRIS-E, in accordance with an amplification of chromosome 13. C4 and C5 were assigned to CRIS-C, in agreement with gains in chromosomes 8 (which contains the proto-oncogene *MYC*) and, for C5, gains in chromosomes 7 (which contains the *EGFR* gene) and 13 (Supplementary Table 1). The lowest immune infiltration (9.7%) was observed in C5, while it increased to ~15% in C1 and C2, 26% in C3 and 31% in C4. C3 and C4 showed a greater fraction of classically activated M1 macrophages and a lack of tumour promoter M2 macrophages. C3 showed greater abundance of B cells, low abundance of T cells and a lack of neutrophils. Lower CD4/CD8 ratios were seen in C4 and C5 (Fig. 3i).

## Discussion

Increasing numbers of syCRCs are identified as early diagnosis technologies improve. syCRCs have distinguishing features to solitary CRCs, with currently no specific guidelines to their management^27,28^. As a rule, the tumour with the highest TNM stage is utilised as a guide for prognosis and clinical management, with lymph node positivity as the most important parameter. The whole-genome analysis of syCRCs in this study highlights how each patient represents a completely distinct scenario. Overall, our results show a high degree of genetic heterogeneity between syCRCs. Synchronous lesions within a patient harbour mainly distinct mutations in the same known CRC genes, although overlaps of few known driver mutations, such as *BRAF* V600E, did occur. Indeed, the presence of the same *BRAF* V600E mutation in all tumours in patient C is an interesting finding, in particular as the mutation also occurs in the metachronous tumour C5. *BRAF* mutation in the setting of MSI is strongly associated with sporadic CRC. In patient C, this shared mutation could either suggest a common tumour origin or is a striking example of convergent evolution in tumour development, likely arising via the serrated BRAF pathway from sessile serrated polyp precursors. However, overall our results suggest syCRCs have a tendency to originate independently, while often accessing the same mutational processes^29,30,32^. In terms of the temporal development of the tumours, for the older patients B (79 years of age) and C (70 years of age) there are no records of prior endoscopy or biopsies prior to the index admission. Therefore, the tumours in these patients may have formed at various time points over the years. Patient A had a longstanding history of ulcerative colitis and was enrolled in a programme of routine endoscopic evaluation for surveillance of dysplasia. The finding of dysplasia in patients with ulcerative colitis is a known predictor of risk of subsequent development of CRC and the tumours are likely to have developed synchronously.

DNA analysis of gut microbial organisms showed that the distribution of *Fusobacteria* is in concordance with previous observations reporting an increased abundance of *F. nucleatum* in *BRAF* mutant, hypermutated, MSI tumours^10^. Analysis of the microbiome associated with each tumour in patient A revealed reduced diversity of the gut microbiota, which could be reflective of a dysbiosis related to the patient’s history of ulcerative colitis^34^, although these results could have been influenced by the administration of an antibiotic bowel preparation in this case. A previous study has shown differences in immune cell scoring between synchronous tumour pairs^29^. Immune score quantification has been validated as a prognostic marker of risk of recurrence in colon cancer, with the quality and density of the immune infiltrate affected by factors including the pre-existing tumour microenvironment, the tumour genetics and the gut microbiome^35^. Our findings reveal differences in microbial and immune composition between synchronous tumours, further highlighting the complexity inherent in these tumours. In addition, recent findings have suggested that patients with *Fusobacterium*-positive tumours could benefit from the administration of antibiotics^10^, although research into the efficacy of microbial-targeted treatments is still in its early stages.

Transcriptomic-based CRC molecular subtyping has revealed clinical and prognostic associations of CRC subtypes^25^. We show heterogeneity in molecular subtype classification of synchronous tumours in two out of three patients. Transcriptome analysis assigned both of Patient A’s tumours to the same molecular subtype (CMS4/CRIS-B). Relevant shared features between CMS4 and CRIS-B include high CIN, TGF-β activation and epithelial-to-mesenchymal transition (EMT), and are associated with poorly and de-differentiated tumours with a stromal-mesenchymal phenotype. This TGF-β/EMT immune phenotype is consistent with the patient’s history of ulcerative colitis. In addition, the shared molecular subtype of A1 and A2 is not unexpected, as histopathological review of the whole colon showed background low-grade dysplasia. However, tumours in patients B and C fell into different subtypes, highlighting the heterogeneity in these patients. Of the nine MSI tumours included in this study, only one, C5, was assigned to the CMS1 group, proposed as the ‘MSI-immune’ group^25^. Tumour C5 was the metachronous primary (occurred >6 months later) and the only poorly differentiated MSI tumour (60% solid growth; Table 1), potentially contributing to the inter-tumour transcriptomic heterogeneity in this patient. A further three of the MSI tumours (B2, B3 and B4) were classified as CRIS-A: the sub-group proposed to include MSI tumours^26^. This highlights the difficulty of applying very defined classification systems to individual tumours with different driver mutations and genomic alterations. As immunotherapy is currently only recommended for MSI-H deficient MMR tumours^36^, deeper investigation into how the transcriptional patterns in syCRC MSI tumours correspond to response to immunotherapy is warranted.

Overall, we found variation in common biomarkers, such as *BRAF* and *KRAS*, for targeted therapies^21^ and also in other features that might impact or predict treatment response^20^. In Patient A’s tumours (both MSS), the *KRAS* wild type status and increase in *EGFR* copy number make them candidates for treatment with the anti-EGFR therapy Cetuximab^37,38^ if they should recur. The CMS4 and CRIS-B classifications for these tumours predict worse relapse-free and overall survival for this patient. In Patient B, the MSI samples B1–B4 show potential to respond to immunotherapy, while this option would most likely have no effect on the MSS B5 cancer, in which, although we see an amplification of *EGFR*, we also find a *KRAS* mutation which therefore excludes anti-EGFR therapy as a treatment option^39^. CRIS-A is associated with a lack of response to anti-EGFR therapy, which agrees with the normal copy state of *EGFR* in B2, B3 and B4. Anti-metabolic therapies have been suggested for CRIS-A subgroups^26^. In Patient C, all five tumours show potential to benefit from similar treatments, namely BRAF inhibition and, especially in the case of C3 and C4, immunotherapy. The specific role of BRAF inhibition in MSI CRC, however, remains to be determined^21^. CRIS-C is associated with Cetuximab-sensitive tumours, proposing this therapy as a viable option for C4 and C5, while CRIS-E predicts poor response to anti-EGFR treatment in C2.

In conclusion, to the best of our knowledge, we have conducted the first WGS study of syCRCs. This has allowed us much broader analytic resolution outside the exome in terms of identifying shared/private mutations in each tumour, and the ability to determine microbial composition in the tumour and matched normal samples. In addition we have been able to conduct analysis of structural variation, and show the SV heterogeneity in patients’ tumours. Furthermore, we have conducted matched RNA-seq analysis of multiple syCRCs. This has facilitated both CRC consensus molecular subtyping and immune composition analysis.

Previous studies have, in all but a few patient cases, conducted exome analyses of paired synchronous tumours from patients. In two of our patient case studies we have analysed five tumours from each patient to construct a much richer depiction of the overall genomic and transcriptomic heterogeneity in multiple syCRCs. While our findings may not have impacted on the standard of care treatment for these patients, compared with previous studies we have identified heterogeneity in current and emerging CRC biomarkers, which may have to be factored into clinical decision-making for patients in the future.

In summary, our study highlights heterogeneity in genomic, transcriptomic, microbial and immune CRC biomarkers in syCRC patients, which could have strong implications for therapeutic management, and requires thorough and careful examination.

## Methods

### Clinical samples

Twelve tumour samples were collected from three treatment naive sporadic CRC patients (Patient A, Patient B and Patient C) at St. Vincent’s University Hospital (SVUH) in Dublin. Fresh tumour and normal tissue were obtained from surgical resection specimens, with normal tissue blocks taken some distance from the invasive tumours. Klean Prep (which includes macrogol) was administered as a routine pre-operative bowel preparation in two of three cases (patients A and B). Patient C did not have bowel prep as was operated on as an emergency due to bowel obstruction. Antibiotic bowel preparation was administered in one (patient A, ciprofloxacin and metronidazole) along with pre-operative hydrocortisone. In each case, the tumours were subjected to the SVUH routine screening protocol for MSI testing. Based on the screening protocol the clinician decides whether to request germline testing or not. In each case germline testing was determined not necessary based on immunohistochemical MSI and BRAF (real time PCR) results in conjunction with clinical history. Adjacent healthy tissue, subjected to pathological quality control, was additionally sampled from each patient to provide a reference of the patient’s normal genome. Written informed patient consent was obtained by the Centre for Colorectal Disease in SVUH and the study was approved by the SVUH Research Ethics Committee. Tumours were classified according to latest American Joint Committee on Cancer (AJCC) TNM system (AJCC 8th Edition: Colorectal Cancer). All samples were stored at −80 °C. Clinicopathological data were available for all patients and are provided in Table 1.

MSI was assessed using IHC for MMR proteins, *MLH1* (BD Bioscience, clone G168-728), *PMS2* (BD Biosciences, clone A16-4), *MSH2* (Calbiochem, clone FE11) and *MSH6* (BD Biosciences, clone 44). IHC was performed on the automated Leica BOND immunostainer.

### DNA and RNA extraction

DNA and RNA extraction from frozen tissue samples was performed at SVUH Dublin.

#### DNA

About 30 mg (2 mm^3^) of frozen tissue was placed into a screw cap vial preloaded with 1.4 mm ceramic beads (Cambio) and samples were homogenised using the Precellys 24 tissue homogeniser (Bertin Instruments) for 20 s at 5500 rpm. Subsequently, samples were incubated at 55 °C in a water bath for 2 h, vortexing the samples every 20 min. DNA isolation was carried out with the *E.Z.N.A*.^®^ Tissue DNA Kit (Omega Bio-Tek) as per the manufacturer’s protocol. Purity was assessed using the NanoDrop noting the A_260/280_ > 1.8. Samples were run on an agarose gel 1% to check for degradation and RNA contamination. Fluorimetric quantification was performed with the Qubit dsDNA HS assay kit (Invitrogen).

#### RNA

About 30 mg (2 mm^3^) of frozen tissue was placed into chilled prefilled tubes with beads (Precellys^®^ Ceramic kit 2.8 mm, reinforced) with 1 ml of lysis buffer. Samples were homogenised at 4 °C using the Precellys 24 at 5500 rpm, 10 s ×2. RNA isolation from snap frozen tissue samples was carried out with the *E.Z.N.A*.^®^ Total RNA kit I (Omega Bio-Tek). RNA purity was assessed using the NanoDrop noting the A_260/280_ > 2. Samples were then run on the Bioanalyzer 2100 (Agilent) and only samples with RNA integrity number > 7 were used for sequencing.

### Whole-genome sequencing

For each patient, all synchronous tumours and a matched normal tissue sample were selected for WGS. Paired end sequencing reads (151 bp) were generated using Illumina HiSeq X sequencing technology, yielding ~×60 coverage per sample. Sequences were aligned to the human reference genome (GRCh37/ hg19) using BWA^40^. PCR duplicates were marked using Picard Tools (http://broadinstitute.github.io/picard) and InDel realignment and base quality recalibration were conducted with the Genome Analysis Toolkit (GATK) v3 (http://www.broadinstitute.org/gatk).

### RNA-sequencing

RNA was isolated from all tumours in each patient and subjected to RNA-sequencing analysis. Sequenced reads were aligned to the human reference genome (GRCh37) using STAR^41^. SNP calling was conducted according to the Broad Institute Best Practices pipeline (https://gatkforums.broadinstitute.org/gatk/discussion/3892/the-gatk-best-practices-for-variant-calling-on-rnaseq-in-full-detail).

### Mutation discovery

Somatic mutations were identified by comparing each tumour sample with adjacent healthy colorectal tissue as a matched normal.

### Substitutions

SNVs were identified with mutation calling algorithms MuTect v1^42^ and Strelka v1^43^. We used BEDTools^44^ to intersect their outputs, and only retained mutations found by both callers. These were further intersected with the dbSNP list of common variants (https://www.ncbi.nlm.nih.gov/SNP/) to exclude potential germline variations. To ensure that no cancer-associated variations were removed, mutations reported in the COSMIC database (https://cancer.sanger.ac.uk/cosmic) were previously excluded from the dbSNP list.

We calculated the variant allele frequency (VAF) of each SNV and further validated mutations by only keeping the ones that met the following parameters: normal alternate allele ≤ 1, minimum combined depth = 20, minimum alternate depth = 2 and minimum VAF = 0.05.

### InDels

InDels were identified with Strelka v1^43^ and filtered from potentially germline variants in the same way as the substitutions (see above).

### Structural variants

SVs (deletions, tandem duplications, inversions, translocations) were identified using DELLY v0.7.9^45^.

### Copy number alterations

CNAs were identified using the R package FACETS v0.5.14^46^ and visualised with the R package copy number v1.24.0^47^.

### Gene annotation and driver analysis

The genic location and functional impact of SNVs, InDels and SVs was annotated using the Ensembl Variant Effect Predictor (VEP) v97^48^. Known driver genes together with MMR and HR genes were searched for causative mutations in all samples. This was done through the Cancer Genome Interpreter^49^ https://www.cancergenomeinterpreter.org/home and VEP^48^. The VAF of each identified driver was calculated to establish its prevalence. CNAs were annotated using the annotate_variation function implemented by ANNOVAR v2018Apr16^50^ and searched for drivers based on known CRC-associated somatic gene CNAs^51^. The relevance of each putative driver CNA was estimated through its median log-ratio, which was provided by the FACETS analysis.

### Mutations overlap with Venn diagrams

The overlap of SNVs, InDels and SVs between the tumours within a patient was calculated and visualised with Venn diagrams using the R package VennDiagram v1.6.20^52^.

### Mutational signature analysis

Mutational signature analysis was performed to inform on the exposures and biological history of a cancer. Mutational signatures were identified from SNVs using the R package deconstructSigs v1.8^53^ based on the pan-cancer catalogue of 30 signatures referenced in the COSMIC database (https://cancer.sanger.ac.uk/cosmic/signatures).

### Gut microbiome analysis

Tumour and normal tissue-associated gut microbiota were identified from DNA data using PathSeq v2.0^54^, available from the GATK v4 (http://www.broadinstitute.org/software/pathseq/).

### RNA-seq data: quantification of gene expression, normalisation and gene ID conversion

Gene counts of tumour samples were generated from RNA-seq data using Kallisto^55^. Normalisation of RNA counts was performed with DESeq2 v1.24^56^, using rlog and the ‘~patient_id’ formula. Ensembl gene IDs were mapped to Entrez and Symbol IDs using biomaRt v2.40^57^.

### Molecular subtyping and tumour immune infiltration

Molecular subtyping of tumours based on gene expression profiles was performed using two R implemented classification systems: the CMS classifier v1^25^, and the CRIS classifier v1^26^.

Tumour immune contexture was analysed for each sample applying the quanTIseq computational pipeline^58^, which uses RNA-seq data to quantify the fractions of ten immune cell types (B cells, classically activated macrophages M1, alternatively activated macrophages M2, neutrophils, natural killer cells, CD4^+^ T cells, CD8^+^ T cells, regulatory T cells, monocytes and dendritic cells) in heterogeneous tissues.

### Reporting summary

Further information on research design is available in the Nature Research Reporting Summary linked to this article.

## Supplementary information

Supplementary Information

Reporting Summary

## Data Availability

Somatic sequence data generated and analysed during the current study have been deposited at the European Genome-phenome Archive (EGA), which is hosted by the EBI and the CRG under study accession number EGAS00001004413. Data are available on request upon publication from the EGA database by contacting the data access committee (Genomic Oncology Research Group DAC: EGAC00001001585) assigned for this project. Data are restricted due to reasons of patient confidentiality.

## Code availability

The data and the results generated in this study were obtained without using any custom code.
