# Supplementary material for: Personalised mapping of tumour development in synchronous colorectal cancer patients
Source: NPJ Genom Med. 2020 Jul 3;5:27. doi: 10.1038/s41525-020-0134-3 (PMC7335056; doi:10.1038/s41525-020-0134-3)
Supplement: Supplementary file 1 — Supplementary Information [file 41525_2020_134_MOESM1_ESM.pdf]

Supplementary Table 1. Mutation burdens (SNVs, InDels, SVs), chromosomal instability status (CIN), proportion of MMR-deficiency related signatures (Sig. 6, Sig. 15, Sig. 20, Sig. 26) and relative MS status (MS), together with evidence of *Fusobacterium nucleatum* (Fuso) and subtype classification (based on CMS classifier and CRIS classifier) are reported for each tumour sample in patient A (A1 and A2), patient B (B1 to B5) and patient C (C1 to C5).

Tumour	SNVs	InDels	SVs	CIN	MMR_sigs	Fuso	MS_status	CMS	CRIS
**A1**	15839	1372	187	High	0	No	MSS	CMS4	CRIS-B
**A2**	11700	253	37	High	0	No	MSS	CMS4	CRIS-B
**B1**	128624	99165	82	Low	0.57	Yes	MSI	CMS2	CRIS-E
**B2**	137831	104406	69	Low	0.45	Yes	MSI	NA	CRIS-A
**B3**	150129	107202	102	Low	0.6	Yes	MSI	NA	CRIS-A
**B4**	220430	155498	139	Low	0.35	Yes	MSI	CMS3	CRIS-A
**B5**	29013	2543	144	High	0	Yes	MSS	CMS2	CRIS-E
**C1**	226130	105426	82	Low	0.71	Yes	MSI	CMS4	CRIS-D
**C2**	177103	128273	58	Low	0.29	Yes	MSI	CMS2	CRIS-E
**C3**	172029	95837	83	Low	0.33	Yes	MSI	NA	CRIS-D
**C4**	130917	95516	217	Low	0.37	Yes	MSI	NA	CRIS-C
**C5**	219246	165272	204	Low	0.39	Yes	MSI	CMS1	CRIS-C

Supplementary Figure Legends

Supplementary Figure 1. (A) A Venn diagram showing the overlap of InDels between tumours. (B) A Venn diagram showing the overlap of SVs between tumours. (C) Tumours mutational profiles of total SNVs, as defined by the weighted contributions of each input reference signature from COSMIC. (D) Values of tumour ploidy and tumour purity, as estimated by FACETS.

Supplementary Figure 2. (A) A Venn diagram showing the overlap of InDels between tumours. (B) A Venn diagram showing the overlap of SVs between tumours. (C) Tumours mutational profiles of total SNVs, as defined by the weighted contributions of each input reference signature from COSMIC. (D) Values of tumour ploidy and tumour purity, as estimated by FACETS.

Supplementary Figure 3. (A) A Venn diagram showing the overlap of InDels between tumours. (B) A Venn diagram showing the overlap of SVs between tumours. (C) IGV overview of the *BRAF* V600E mutation. (D) IGV overview of the *MSH6* gene. (E) Tumours mutational profiles of total SNVs, as defined by the weighted contributions of each input reference signature from COSMIC. (F) Values of tumour ploidy and tumour purity, as estimated by FACETS.

Supplementary Figure 1

A

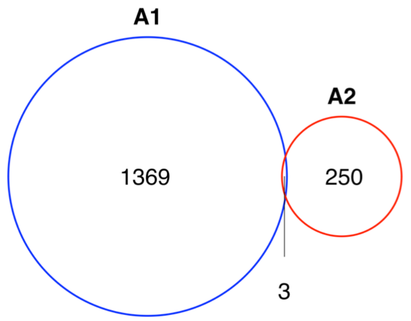

B

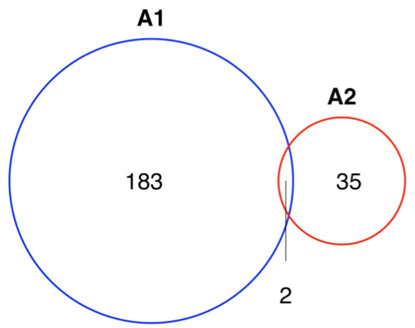

C

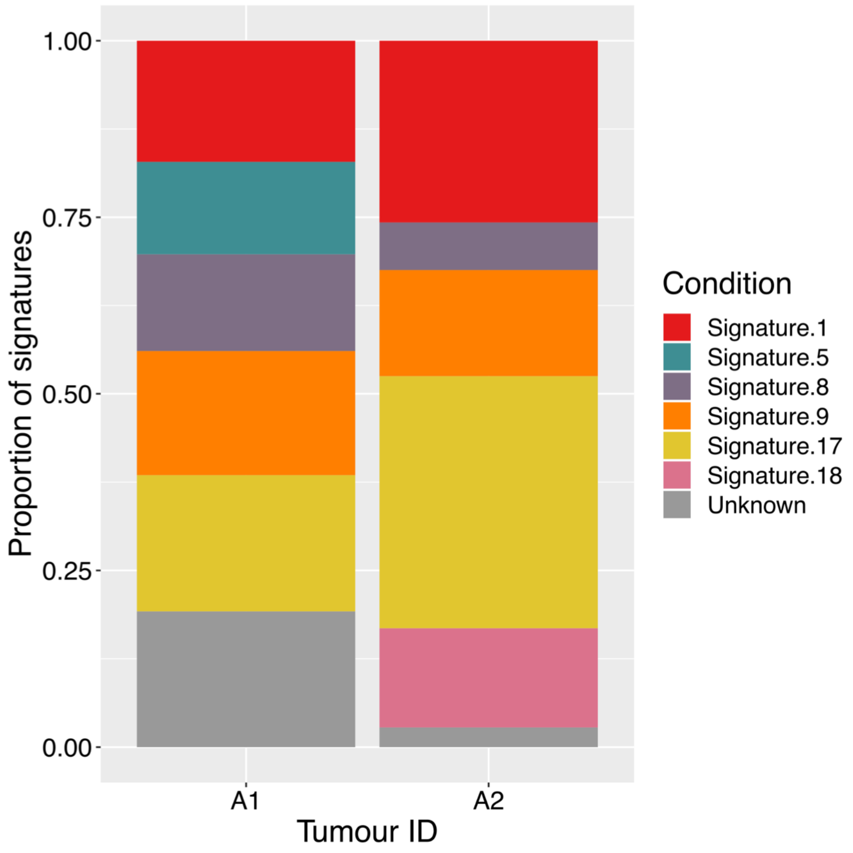

D

	Tumour ploidy	Tumour purity
A1	3.58	0.56
A2	2.2	0.38

Supplementary Figure 2

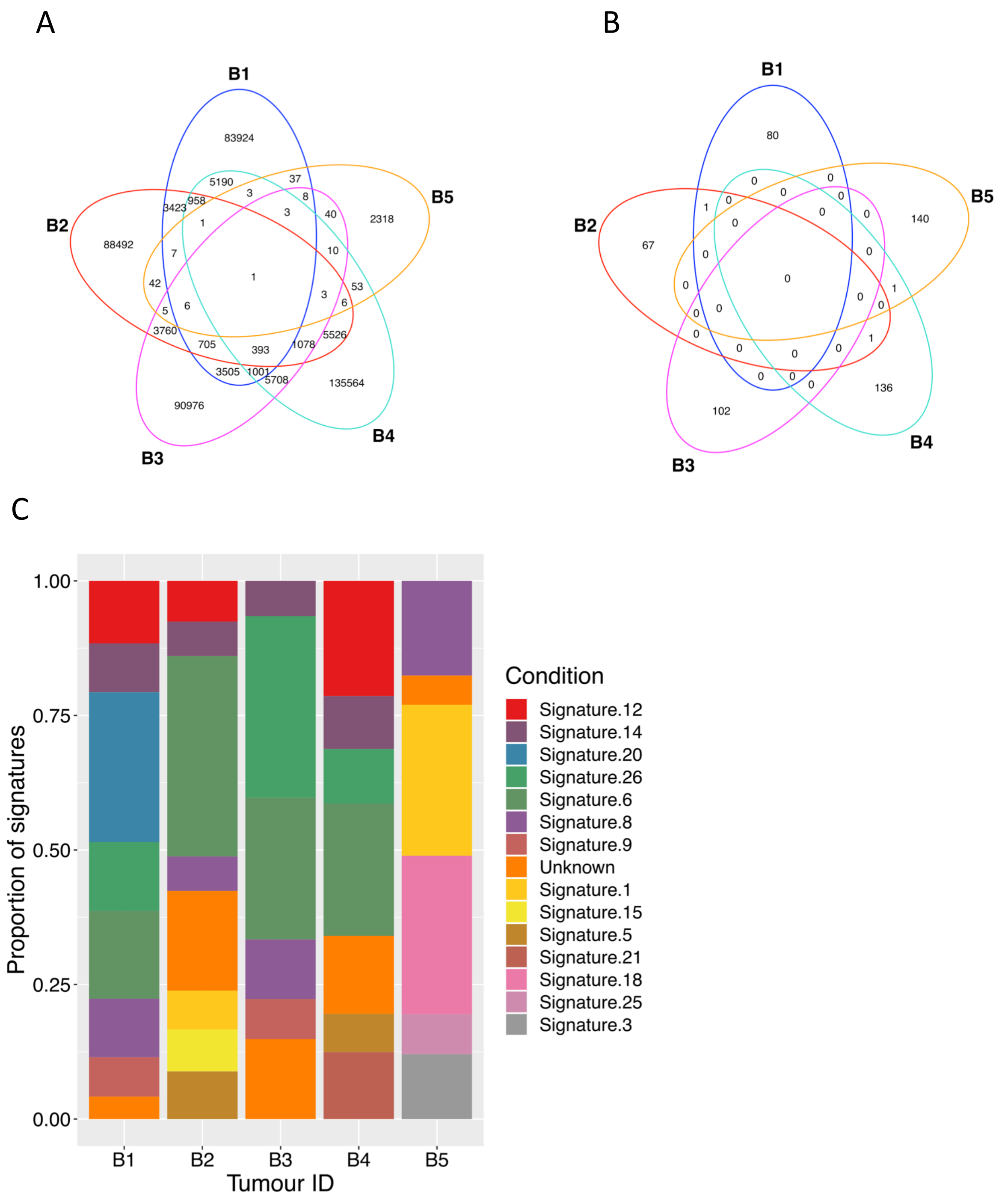

D

	Tumour ploidy	Tumour purity
B1	2	0.3
B2	1.95	0.48
B3	2	0.72
B4	1.98	0.71
B5	2.13	0.8

Supplementary Figure 3

A

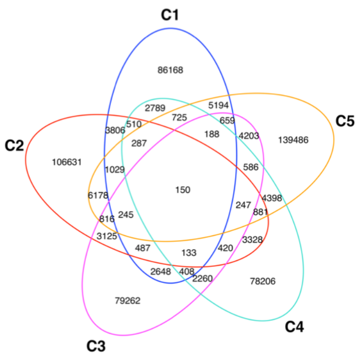

B

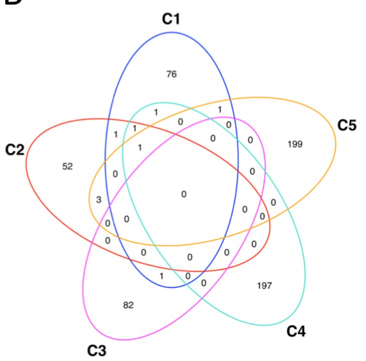

C

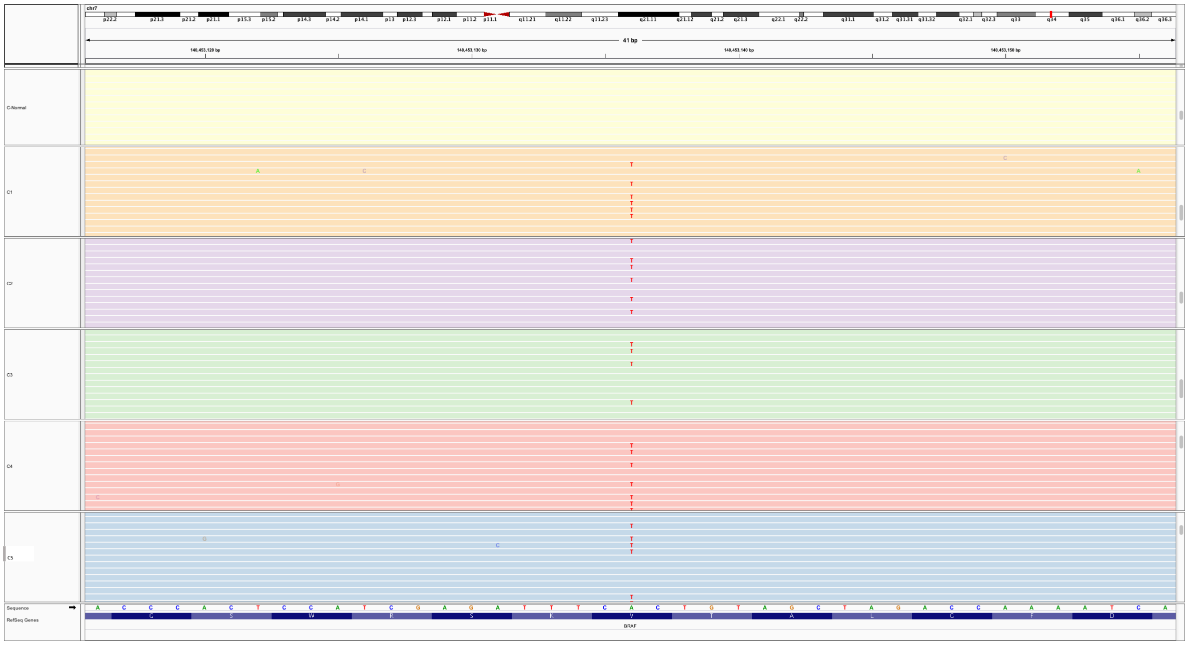

D

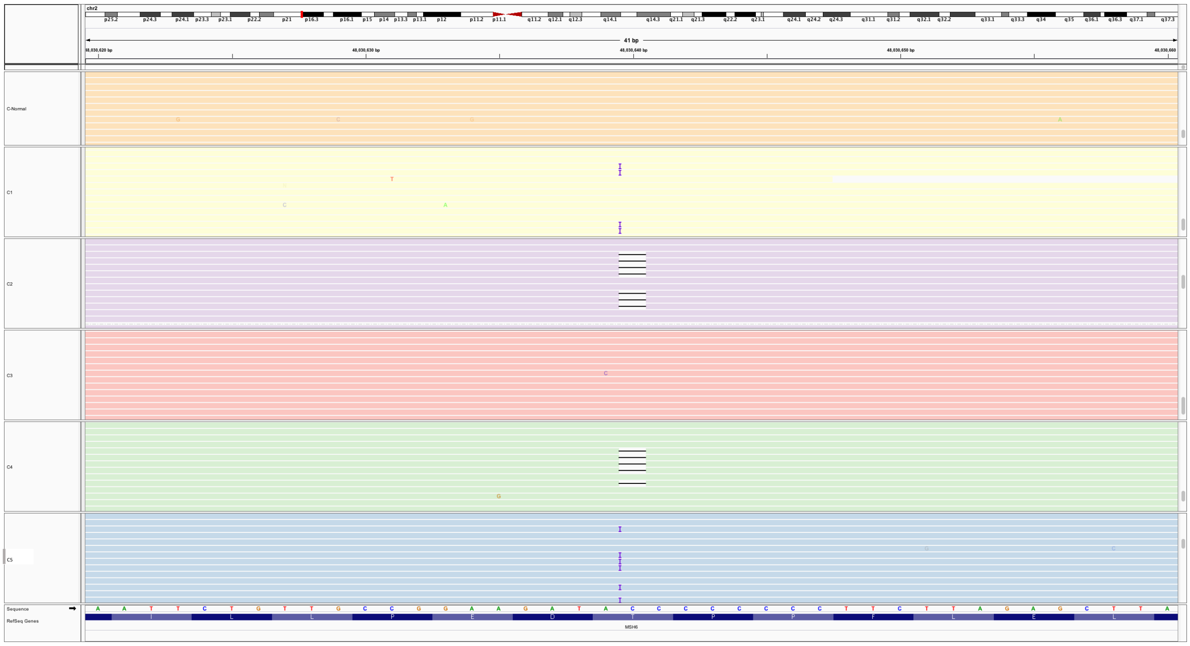

E

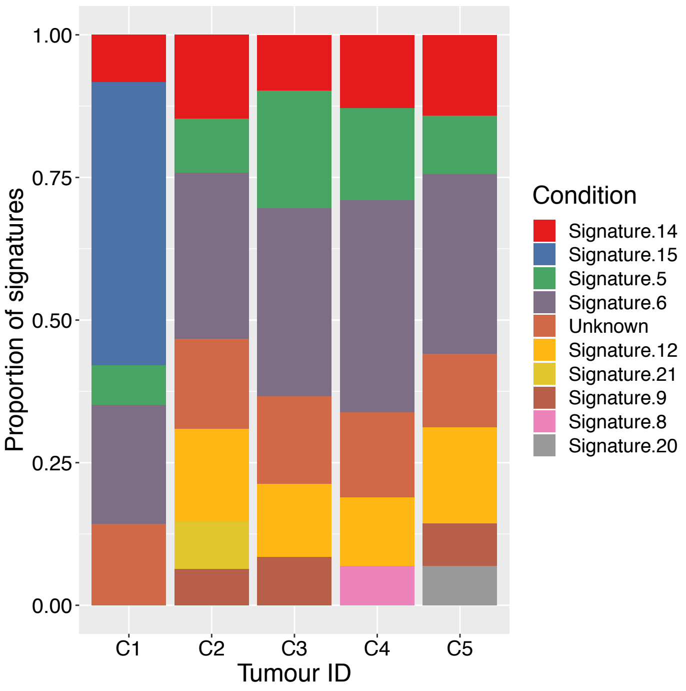

F

	Tumour ploidy	Tumour purity
C1	2.03	0.59
C2	2.06	0.73
C3	2.13	0.35
C4	2.02	0.72
C5	2.13	0.61
